# Constitutive Neutrophil Apoptosis: Regulation by Cell Concentration via S100 A8/9 and the MEK – ERK Pathway

**DOI:** 10.1371/journal.pone.0029333

**Published:** 2012-02-17

**Authors:** Mizhir Atallah, Alon Krispin, Uriel Trahtemberg, Sandrine Ben-Hamron, Amir Grau, Inna Verbovetski, Dror Mevorach

**Affiliations:** The Laboratory for Cellular and Molecular Immunology, Department of Medicine, Rheumatology Research Centre, Hadassah-Hebrew University Medical Center Jerusalem, Jerusalem, Israel; University of Illinois at Chicago, United States of America

## Abstract

Programmed cell death (PCD) is a fundamental mechanism in tissue and cell homeostasis. It was long suggested that apoptosis regulates the cell number in diverse cell populations; however no clear mechanism was shown. Neutrophils are the short-lived, first-line defense of innate immunity, with an estimated t = 1/2 of 8 hours and a high turnover rate. Here we first show that spontaneous neutrophil constitutive PCD is regulated by cell concentrations. Using a proteomic approach, we identified the S100 A8/9 complex, which constitutes roughly 40% of cytosolic protein in neutrophils, as mediating this effect. We further demonstrate that it regulates cell survival via a signaling mechanism involving MEK-ERK via TLR4 and CD11B/CD18. This mechanism is suggested to have a fine-tuning role in regulating the neutrophil number in bone marrow, peripheral blood, and inflammatory sites.

## Introduction

Programmed cell death (PCD) is a fundamental mechanism in tissue and cell homeostasis, long thought to regulate cell number in diverse populations. In developmental biology, PCD provides an efficient mechanism for eliminating unwanted cells, as shown in studies with the nematode Caenorhabditis elegans [1]. Of the 1090 somatic cells formed during the development of an adult hermaphrodite, exactly 131 die, demonstrating the morphological features of apoptosis. In the mammalian immune system, clonal triggering results in lymphocyte proliferation followed by activation-induced cell death that ensures T cells will return to their original concentration, adding only a negligible number of memory cells [2].

Few studies in the human immune system have questioned the mechanism of constitutive leukocyte blood concentration regulation via PCD. In humans, neutrophils are the short-lived, first-line defense of innate immunity, with estimated t = 1/2 of 8 hours and a high turnover rate [3]. These cells, which represent 50–60% of the circulating leukocytes in normal conditions, are considered terminally differentiated cells. They are rapidly recruited and accumulate in the early phase of inflammation.

Under normal physiological conditions, absent triggers initiating neutrophil exit from the blood pool into tissues, human blood neutrophil counts are determined by the balance between neutrophil hematopoiesis, neutrophil mobilization from the bone marrow, the marginal pool, spontaneous constitutive PCD, and phagocytosis of apoptotic neutrophils. Human neutrophils are generated in bone marrow, mainly under the control of G-CSF and additional cytokines and growth factors, including M-CSF, GM-CSF, IL-6, IL-3 (reviewed by Metcalf [4]), and possibly IL-17 and IL-23 [5], as well as IL-22. Martin et al., and others, suggested that neutrophils are retained in the bone marrow through interaction of CXCL12 (stromal derived factor 1, or SDF-1) with its receptor CXCR4 [6]–[7]. In the blood pool, neutrophils circulate until they are marginated or develop morphological changes due to PCD, and are cleared by Kupfer cells in the liver [8] or by bone marrow macrophages [9].

Various cytokines regulate blood neutrophil counts. Catecholamines mobilize the marginal pool [10], while G-CSF, IL-17, and IL-23 [5] may modify counts via bone marrow. Yet, the major regulator of blood neutrophil counts and neutrophils at inflammatory sites is PCD, which allows only a short neutrophil life span, modulated according to specific needs, such as inflammation.

Molecular events leading to this short neutrophil lifespan are better understood now. An important regulator of neutrophil apoptosis seems to be the Bcl-2 protein family, and a major event in spontaneous neutrophil PCD appears related to loss of anti-apoptotic proteins. Bcl-2 is lost during neutrophil differentiation, and not expressed by peripheral blood neutrophils [11]–[14]. Bcl-xl is also expressed by neutrophils, and expression decreases upon TNFα-mediated apoptosis [14]. Mcl1 declines as neutrophils undergo spontaneous apoptosis, and is upregulated when cells are exposed to survival factors such as GMCSF, butyrate, ILβ, and LPS [12]. A1, expressed in neutrophils at the mRNA level [13], [15], is also upregulated by survival factors GCSF and LPS [15]. Furthermore, neutrophils do not express survivin, an inhibitor-of-apoptosis protein (IAP), unless expression is induced by GCSF and GMCSF or in inflammatory conditions [16]. In contrast, pro-apoptotic proteins Bax, Bak, and Bad are constitutively expressed [13], [14], [17].

The extrinsic pathway may also be involved. Neutrophils constitutively express Fas and Fas ligand, although the importance of Fas-induced death remains controversial [11], [18]–[20]. In addition, neutrophil life expectancy can be modulated by various mediators (G-CSF, GM-CSF, IL1β, IFN-gamma, and LPS [21]–[25]) or functional events such as transmigration across the endothelium [26].

Despite our growing understanding of molecular control of neutrophil PCD, it remains unclear how specific neutrophil blood concentration is maintained. We hypothesized that cell concentration might serve as a neutrophil count regulator via a feedback mechanism affecting PCD. We examined the in vitro effect of cell number on spontaneous neutrophil PCD, and demonstrated that neutrophil PCD is strongly influenced by cell concentration. We hypothesized that secreted molecules mediate this effect, and used a proteomic approach, which showed that constitutive spontaneous neutrophil PCD is regulated by cell concentration through molecules released during neutrophil PCD. We further show that the S100 A8/9 complex is a primary regulatory molecule regulating cell survival via CD11b/CD18 and TLR4, and through a signaling mechanism involving MEK-ERK.

## Materials and Methods

### Materials

Cell culture medium consisted of RPMI 1640 (Invitrogen-Gibco, NY) supplemented with 1% L-glutamine and 1% penicillin/streptomycin (Biological Industries, Kibbutz Beit-Haemek, Israel). The APOPTEST-FITC Kit containing Annexin V-FITC was obtained from Nexins Research B.V. (Hoeven, The Netherlands) or from MBL International (Cambridge, MA). Propidium iodide (PI) was from Molecular Probes (Eugene, OR). Recombinant proteins S100A8 and S100A9, and blocking antibodies were generously supplied by Dr. Philippe A. Tessier. **(**Laval University, Quebec, Canada). The pan-caspase inhibitor zVAD-fmk was purchased from R&D systems (Minneapolis, MN).

Mouse anti-human CD36-PE, mouse anti-human CD11b-PE, and isotype control IgG1-PE were obtained from Serotec (Oxford, UK), and isotype control mouse IgM-PE from Dako (Glostrup, Denmark).

DiOC6(3) (3,3-dihexyloxacarbocyanine iodide) was purchased from Sigma-Aldrich (St. Louis, MO). MEK inhibitor PD98059 was purchased from Cell Signaling (Danvers, MA).

Antibody against the complex S100A8/9 for flow cytometry analysis was purchased from Abcam (Cambridge, MA).

For blocking assays, rabbit polyclonal anti-S100A9 and anti-S100A8 were generously supplied by Dr Philippe A. Tessier, and blocking antibody against CD11b (BioLegend, San Diego, CA) was used at different dilutions as indicated. Rabbit serum was obtained from Jackson Laboratories (Jackson ImmunoResearch, West Grove, PA).

### Cell isolation and culturing

Blood neutrophils were isolated from fresh buffy coats obtained from healthy donors. RBCs were sedimented by adding 6% hetastarch in 0.9% NaCl solution (Stem Cell Technologies, Vancouver, Canada) and kept at 25°C for up to 45 min. The leukocyte-rich upper layer of the suspension was then collected and centrifuged on a density gradient with Ficoll-Paque (Amersham Biosciences, Uppsala, Sweden). Residual erythrocytes were removed by hypotonic lysis. Neutrophils were maintained in suspension, at different concentrations as indicated, in RPMI 1640 medium, 1% L-glutamine, and 1% penicillin/streptomycin, in 24-well plates at 37°C, in a humidified incubator containing 5% CO_2_. Cells were >95% neutrophils as determined by morphological analysis and >99% viable as determined by Trypan blue dye exclusion

#### Apoptosis assessment

Apoptosis assessment was performed as previously described [27] (For detailed method see – Methods S1).

### Supernatant transfer

Neutrophils were suspended at concentrations of either 0.5×10^6^/ml or 16×10^6^/ml, for different times as indicated, at 37°C/5% CO_2_. After culturing, the supernatants of the high concentration were collected and added to fresh neutrophils from the same donor at concentration of 0.5×10^6^/ml and vice versa. The neutrophils were then allowed to undergo constitutive spontaneous PCD and apoptosis was detected as described.

### Transmission electron microscopy

Cells were fixed in 2% glutaraldedyde in cacodylate buffer (0.1M, PH 7.2–7.4) and washed three times in the same buffer. Cells were then postfixed in 1% OSO4 for 1 h at room temperature, washed in cacodylate buffer, dedydrated in gradient series of ethanol (25%, 50%, 75%, 95%, 100%×2), treated with propylene-oxide for 20 min (2 changes), and embedded in Araldite Resin. Thin sections were prepared with an ultramicrotom and examined at an accelerated voltage of 100KW by transmission electron microscope (CM12, Philips, Eindhoeven, The Netherlands).

#### Proteomic (SDS-PAGE and MS) and Western blotting

Proteomic (SDS-PAGE and MS) and Western blotting were performed as previously described [28] (For detailed method see – Methods S1).

### Transfection

The two cell lines, Chinese hamster ovary (CHO), and CHO stably transfected with complement receptor CR3, were kindly provided by Drs. R.R. Ingalls and D.T. Golenbock, Boston Medical Center, Boston, MA.

### Leukocyte adhesion deficiency patients and controls

LAD patients signed an informed consent approved by the Ethics Committee of Hadassah-Hebrew University Medical Center. Controls consisted of age- and gender-matched healthy donors, who signed an informed consent. Blood (10 ml) from healthy donors and heparinized blood (10 ml) from patients was drawn and evaluated on the same day.

### Detection of intracellular phosphorylated ERK by flow cytometry

For the assessment of phosphorylation of the ERK, freshly isolated neutrophils were treated as indicated and prepared for intracellular staining. Cells were washed with PBS, fixed with 2% formaldehyde for 10 min at 37°C, and permeabilized with 90% methanol for 30 min on ice. Cells then were rinsed and incubated for 10 min at RT in PBS containing 0.5% BSA (for blocking), and stained with either mouse anti-human phospho-p44/42 MAPK alexa fluor 488 (Cell Signaling Technology) or isotype control mouse IgG1 alexa fluor 488 (BioLegend).

### Statistical analysis

The Student's t test and one-way analysis of variance (ANOVA) were used to compare mean data. The Kolmogorov-Smirnov test was used to analyze flow cytometry results. Differences were considered statistically significant for *p*<0.05.

## Results

### The effect of cell concentration on constitutive spontaneous neutrophil PCD

**Figure 1:**
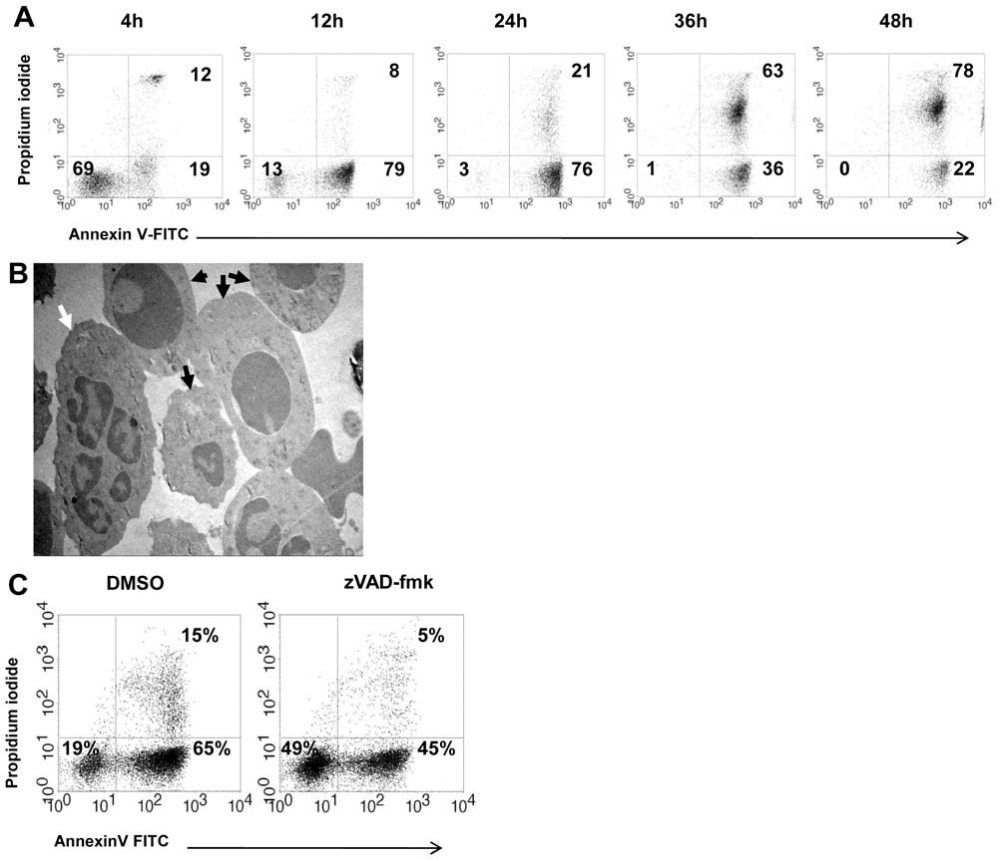
Constitutive spontaneous neutrophil PCD. **A.** Kinetics of spontaneous constitutive neutrophil PCD. Neutrophils were isolated and allowed to undergo spontaneous constitutive PCD at a concentration of 1×10^6^/ml. Samples were obtained at the indicated intervals, and the apoptosis rate was measured using Annexin V-FITC and PI staining. **B.** Transmission electron microscopy (TEM) of spontaneous constitutive neutrophil PCD. Morphology of viable or early apoptotic (white arrow) and apoptotic (black arrows) neutrophils is shown. Apoptotic cells show the typical morphology of condensed cytoplasm and chromatin. Cells were prepared for TEM as described in Materials and methods. **C.** Inhibition of spontaneous constitutive neutrophil PCD by pan-caspase inhibitor zVAD-fmk. Sample dot plots of AnnexinV-PI staining of neutrophils undergoing spontaneous constitutive PCD for 14 h at a concentration of 1×10^6^/ml, in the presence or absence of 20 µM Zvad-fmk. Pan-caspase inhibition rescued cells from apoptotic death and viable cells are increased from 19 to 49% (*p*<0.001). Data are representative of 3 or more experiments.

**Figure 2:**
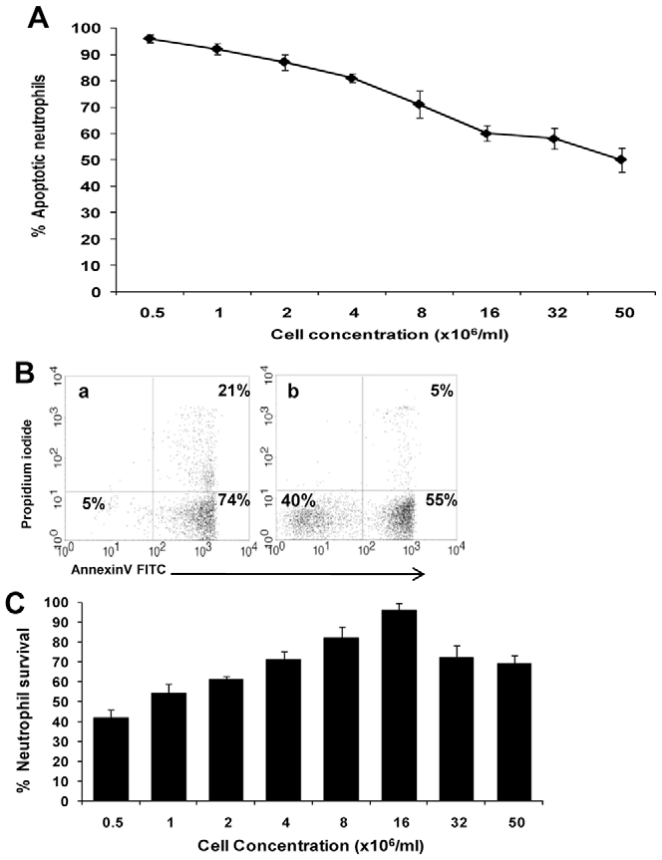
Spontaneous constitutive neutrophil PCD is cell concentration-dependent. **A.** Neutrophils underwent spontaneous constitutive PCD for 12 h at cell concentrations ranging from 0.5×10^6^/ml up to 50×10^6^/ml. (The physiological blood concentration is 2−6×10^6^/ml.) Apoptosis was assessed by AnnexinV-FITC and PI staining, as described in Materials and methods. Data represents the mean ± SD of 3 experiments. **B.** Sample dot plots of AnnexinV-PI staining of neutrophils undergoing spontaneous constitutive PCD for 12 h in (a) 0.5×10^6^/ml, and (b) 16×10^6^/ml. The percentage of viable, early (AnnexinV^+^/PI^−^) and late (AnnexinV^+^/PI^+^) apoptotic cells is indicated within the respective quadrants. **C.** Survival of neutrophils undergoing spontaneous constitutive PCD, based on cell counts. Data presented is the mean ± SD of 3 experiments.

We assumed constitutive spontaneous PCD requires one or more autoregulatory negative feedback mechanisms exerted by dying neutrophils that affect survival rates of the remaining cells (Fig. 1). Without this effect, one might assume that PCD increases at higher concentrations due to competition for nutrition and other ingredients. We designed an experiment to examine the effect of various cell concentrations in the physiological range (peripheral blood neutrophil concentration ∼2−6×10^6^/ml) on the constitutive spontaneous PCD rate. A higher apoptotic cell concentration had a protective anti-apoptotic effect, and the rate of neutrophil PCD was inversely proportional to cell concentration, indicating an anti-apoptotic effect (Fig. 2A). Whereas following 12h of spontaneous constitutive PCD only 5% of cells were still alive at a concentration of 0.5×10^6^ neutrophils/ml, in conditions of 32 fold concentration, at 16×10^6^ neutrophils/ml, more than 40% of the cells were still alive, as shown by Annexin V and PI negative staining (Fig. 2B) and verified by mitochondrial potential loss studies (not shown), both methods of apoptosis detection indicated early apoptosis state. This phenomenon was further emphasized using cell count. There was loss of over 50% of cells through constitutive spontaneous PCD at concentrations of ­­­­0.5x10^6^/ml for 12h (Fig. 2C), and this loss decreased in proportion to increasing cell concentrations up to 16x10^6^/ml. At concentrations above 16x10^6^/ml, there was a decline in cell number and a prodeath effect, probably as a result of competition for nutrition and other ingredients.

Thus, at physiological concentrations of neutrophils, there was an anti-apoptotic effect at higher neutrophil concentrations. We termed this a “community effect” ― neutrophils are encouraged to live longer as their community is enlarged.

### Factors mediating the community effect are present in the supernatant

**Figure 3:**
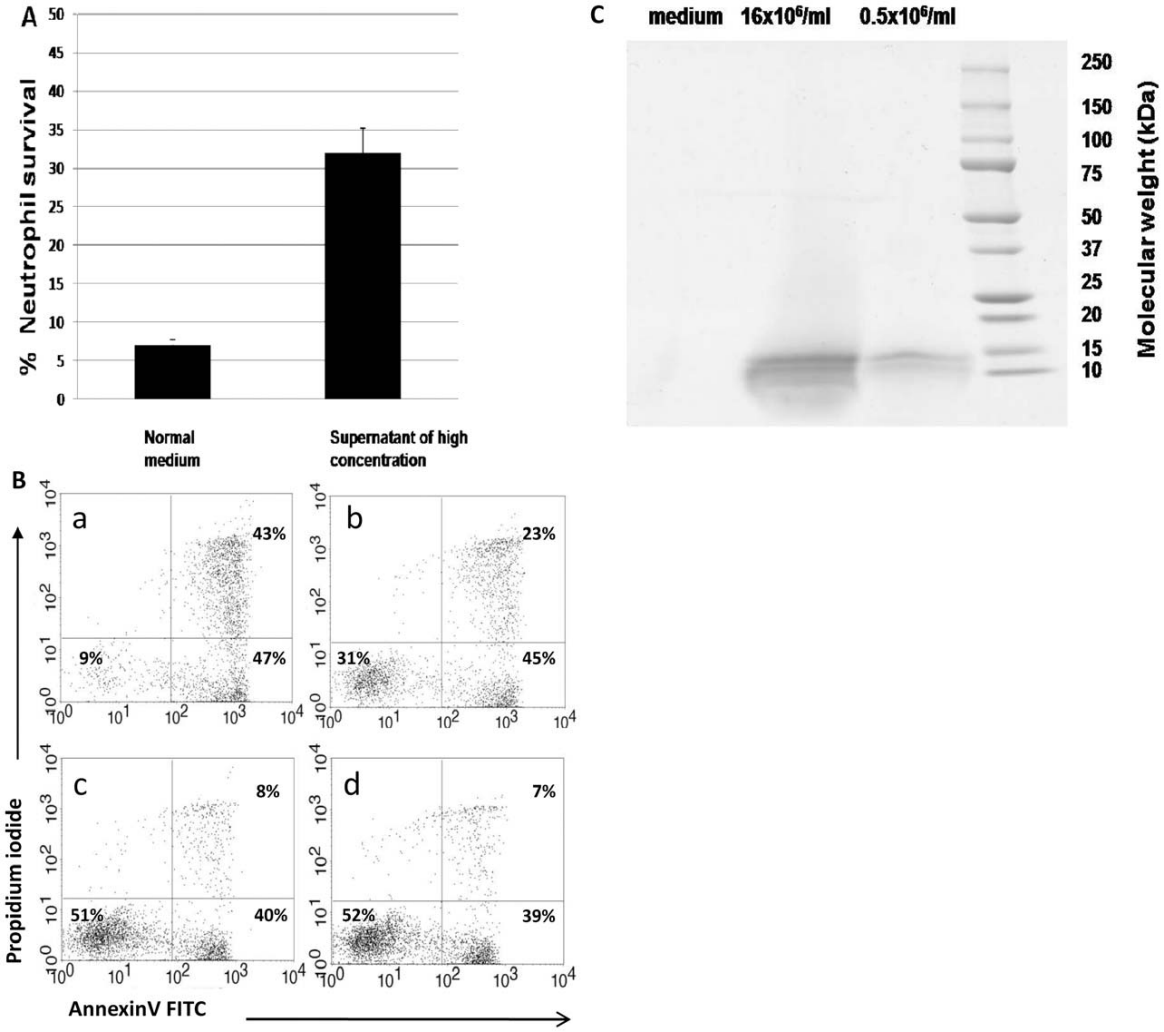
Identification of candidate molecules that induce the community effect. **A.** Supernatant of cells undergoing spontaneous constitutive PCD at high concentrations has rescued cells undergoing spontaneous constitutive PCD from apoptosis at low concentrations, and improved their survival (*p*<0.001). Data is representative of 3 experiments. **B.** Representative dot plot of supernatant transfer assay. Apoptosis was assessed by AnnexinV and PI staining of neutrophils after 12 h of spontaneous constitutive PCD. The transfer assay was performed as follows: neutrophils underwent spontaneous constitutive PCD at low (a, 0.5×10^6^/ml) or high (c, 16×10^6^/ml) concentration for 12 h. The supernatant of the cells incubated at high concentrations was collected and used as media for neutrophils undergoing constitutive spontaneous PCD at low concentrations (b), and the supernatant of the cells incubated at low concentrations was collected and used for neutrophils undergoing constitutive spontaneous PCD at high concentrations (d). **C.** SDS-PAGE of differentially displayed proteins from high- and low-concentration spontaneous constitutive PCD. The supernatants of cells undergoing spontaneous constitutive PCD at high- and low concentrations were collected, and the proteins were purified as described in Materials and methods. Protein samples were electrophorized and stained with Coomassie, as shown. Two proteins, ∼14 KD and ∼10 KD, were identified in the supernatant of cells undergoing spontaneous constitutive PCD at high concentrations (16×10^6^/ml).

In order to determine whether the factor(s) responsible for the community anti-apoptotic effect are found in the supernatant, we exchanged media obtained from low- and high concentrations of constitutive spontaneous neutrophil PCD. The supernatants from high concentrations were collected and used as media for low-concentration constitutive spontaneous neutrophil PCD of the same donor, and the supernatants of low concentrations were used as media for constitutive spontaneous neutrophil PCD at high cell concentration. Media based on high-concentration PCD donors (16×10^6^ cells/ml) rescued cells undergoing constitutive spontaneous neutrophil PCD at low concentration (0.5×10^6^ cells/ml) from apoptosis, as compared with normal medium (Fig. 3A). High-concentration medium increased the rate of viable cells from 9% (upper left panel, Fig. 3B) to 31% (upper right panel, Fig. 3B). In the opposite experiment, low-concentration media had no effect on survival in high cell concentration samples (left and right lower panels, Fig. 3B). All these experiments were based on at least three experiments each and results with Annexin V were verified using mitochondrial potential loss studies.

These results suggest that in the presence of high neutrophil concentrations, anti-apoptotic factor(s) are secreted and provide a survival signal to the viable neutrophil community.

### Proteomic analysis of the supernatants and identification of S100A8 and S100A9 as candidate molecules for mediation of the community anti-apoptotic signal

We used a differentially displayed proteomic approach, as we have previously described [28], to search for secreted factor(s) in the supernatant that may be responsible for the “community effect” on neutrophil survival.

The secreted proteomes of neutrophils undergoing constitutive spontaneous neutrophil PCD at high concentrations (16×10^6^/ml) and low concentrations (0.5×10^6^/ml) were collected and compared by SDS-PAGE and Coomassie staining. Differentially expressed proteins were further analyzed by mass spectrometry (MS). Expression of two proteins, ∼10 KD and ∼14KD, represented the primary difference between the supernatants (Fig. 3C). Both were expressed at significantly greater levels in supernatant of cells cultured at high neutrophil concentrations, versus supernatant of cells cultured at low concentrations, despite protein normalization according to cell number and equal protein loading in the gel. Proteins were further identified by MS as S100A8 and S100A9, as the best candidates for the community effect (Table 1). The experimental approach was designed to use 2D gel; however, 1D gel identified with high certainty two candidate proteins that were chosen for further functional studies.

**Table:** **Table 1.** Proteins identified by mass spectrometry.

Peptide sequence	Protein score (Mowse)	Identified protein	Remarks
MTCKMSQLER **NIETIINTFH QYSVKLGHPD TLNQGEFK**EL VRKDLQNFLK KENKNEK**VIE HIMEDLDTNA DKQLSFEEFI MLMAR**LTWAS HEKMHEGDEG PGHHHKPGLG EGTP	330	S100A9	Reported role in inflammation
**MLTELEKALN SIIDVYHK**YS LIK**GNFHAVY** **RDDLKKLLET ECPQYIRKKG** **ADVWFKELDI NTDGAVNFQE FLILVIK**MAW QPTKKAMKKA TKSS	458	S100A8	Reported role in inflammation
MVHLTPEEKS AVTALWGK**VN VDEVGGEALG RLLVVYPWTQ RFFESFGDLS TPDAVMGNPK** VKAHGKKVLG AFSDGLAHLD NLKGTFATLS ELHCDKLHVD PENFRLLGNV LVCVLAHHFG K**KFTPPVQAA YQK**VVAGVAN ALAHKYH	207	beta globin chain variant	Beta globin chain
VLSPADKTNV KAAWGK**VGAH AGEYGAEALE RMFLSFPTTK** TYFPHFDLSH GSAQVKGHGK KVADALTNAV AHVDDMPNAL SALSDLHAHK LRVDPWNFKL LSHCLLVTLA AHLPAEFTPA VHASLDKFLA SVSTVLTSKY R	94	Chain C, T State Human Hemoglobin [alpha V96w]	Hemoglobin chain
R**RPDFCLEPP YTGPCK**ARII RYFYNAK**AGL CQTFVYGGCR** AKRNNFKSAE DCMRTCGGA	113	Aprotinin	Protease inhibitor (synthetic)

Analysis of proteins identified by mass spectrometry. Sequences corresponding to the identified peptides are bolded. Score was based on molecular weight search (MOWSE) peptide-mass database. The results identified S100A8 and S100A9 as candidate proteins.

### Functional studies suggest that S100A8-9 mediates the community effect

**Figure 4:**
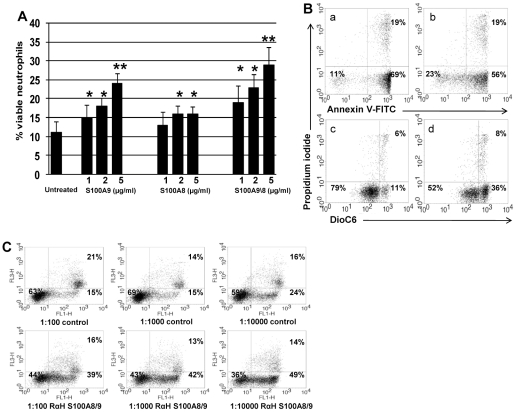
Effect of S100A9 and S100A8 on neutrophil apoptosis. **A.** S100A9 and S100A8 add-in experiments. Varying concentrations of S100A9, S100A8, and the S100A8/9 complex were added to supernatants of neutrophils undergoing spontaneous constitutive PCD at a concentration of 0.5×10^6^/ml. After 10 hours apoptosis was evaluated using Annexin V-PI and mitochondrial staining. Data is presented as mean ± SD of Annexin V-PI staining (* p<0.05, ** p<0.02). **B.** S100A9 and S100A8 add-in experiments. A representative sample of Annexin V-PI and mitochondrial staining. Neutrophils undergoing spontaneous constitutive PCD without (a and c) or with (b and d) the addition of 2 µg/ml S100A8/9. Cells were stained either with Annexin V-FITC (a and b) or with DiOC6 (c and d), as well as PI (a, b, c, d) and assessed by flow cytometry. Dot plots are representative of 6 experiments. **C.** The effect of anti-S100A8 and A9 on add-in experiments. Neutrophils undergoing spontaneous constitutive PCD at a concentration of 0.5×10^6^/ml for 12h, with rabbit polyclonal antibody dilutions of 1∶100, 1∶1000, and 1∶10,000 against S100A9 and S100A8, were then treated with 2 µg/ml S100A8/9 complex. Rabbit serum at the same dilutions of 1∶100, 1∶1000, and 1∶10,000 was used as a control. Apoptosis was assessed using Annexin V-FITC and PI staining. Percentages of viable, early-, and late apoptotic cells are indicated within the respective quadrants. Dot plots are representative of 6 experiments.

We sought to verify S100A8 and A9 mediation of the community anti-apoptotic effect using recombinant human S100A8 and A9 (kindly provided by Philippe A. Tessier, Laval University, Quebec, Canada) added to neutrophils undergoing spontaneous constitutive PCD. Increased S100A9 and S100A8/9 levels had a dramatic rescue effect on the number of neutrophils undergoing spontaneous constitutive PCD at low cell concentrations (* p<0.05), paralleling the rescue effect of the high cell concentration supernatant (Fig. 4A). Survival increased by more than 150%±35% in the presence of 5 µg/ml of S100A8/9, as measured by Annexin V and PI (** p<0.02). The survival effect of S100A9 and the S100A8/9 complex was more prominent compared to S100A9 alone. Only S100A9 had an effect already at a concentration of 1 µg/ml. Nonetheless, S100A8 in higher concentrations of 2 and 5 µg/ml also had a significant rescue effect (Fig. 4A). To further verify the anti-apoptotic effect of S100A8/9, we measured mitochondrial transmemebrane potential as an additional early PCD evaluation, demonstrating a perfect correlation to the Annexin V-PI measurements (Fig. 4B).

We sought to verify whether S100A9 and S100A8/9 effects are blocked with specific antibodies, using rabbit polyclonal antibodies raised against human S100A8 or S100A9. Antisera were used at a range of dilutions between 1/10 and 1/10,000, as indicated. Adding the antibodies reduced neutrophil survival by 30−40% in various dilutions (Fig. 4C), compared to control antibodies, and induced accelerated PCD similar to that seen in spontaneous constitutive PCD in low cell concentration supernatants. Mitochondrial transmemebrane potential as an additional early PCD evaluation, demonstrated a perfect correlation to the Annexin V-PI measurements. This further supports a role for S100A8/9 as the main factor triggering the community anti-apoptotic effect.

### S100A8/9 does not mediate the community effect via McL1, A1, the bcl-2 protein family, or survivin

We examined the expression of different members of the PCD intrinsic pathway, focusing on those known to be important in neutrophil PCD. McL1, an anti-apoptotic member, was expressed by freshly isolated neutrophils (see Figure S3), and expression was still detected during spontaneous constitutive apoptosis. Adding S100A8/9 did not change McL1 expression. A1 protein has also been found to be expressed in neutrophils [13], [15]. We found that A1 was expressed by freshly isolated neutrophils, with expression dramatically decreased during constitutive spontaneous PCD (see Figure S3). Adding S100A8/9 did not prevent decreased A1 expression.

Bclxl was found to be expressed by freshly isolated neutrophils. Expression was neither decreased by constitutive spontaneous PCD, nor increased by adding S100A8/9. We also examined the expression of Bcl2 and survivin proteins. These two proteins were not expressed by freshly isolated neutrophils or during induction of constitutive spontaneous PCD, which is consistent with observations that peripheral blood neutrophils don't express these two proteins [11−14], .

In conclusion, we could not identify an anti-apoptotic mechanism in the main intrinsic pathway players of neutrophil PCD.

### The survival effect of S100A8/9 involves the MEK-ERK signaling pathway, and is partially mediated through TLR4 and the integrin CD11b/CD18

**Figure 5:**
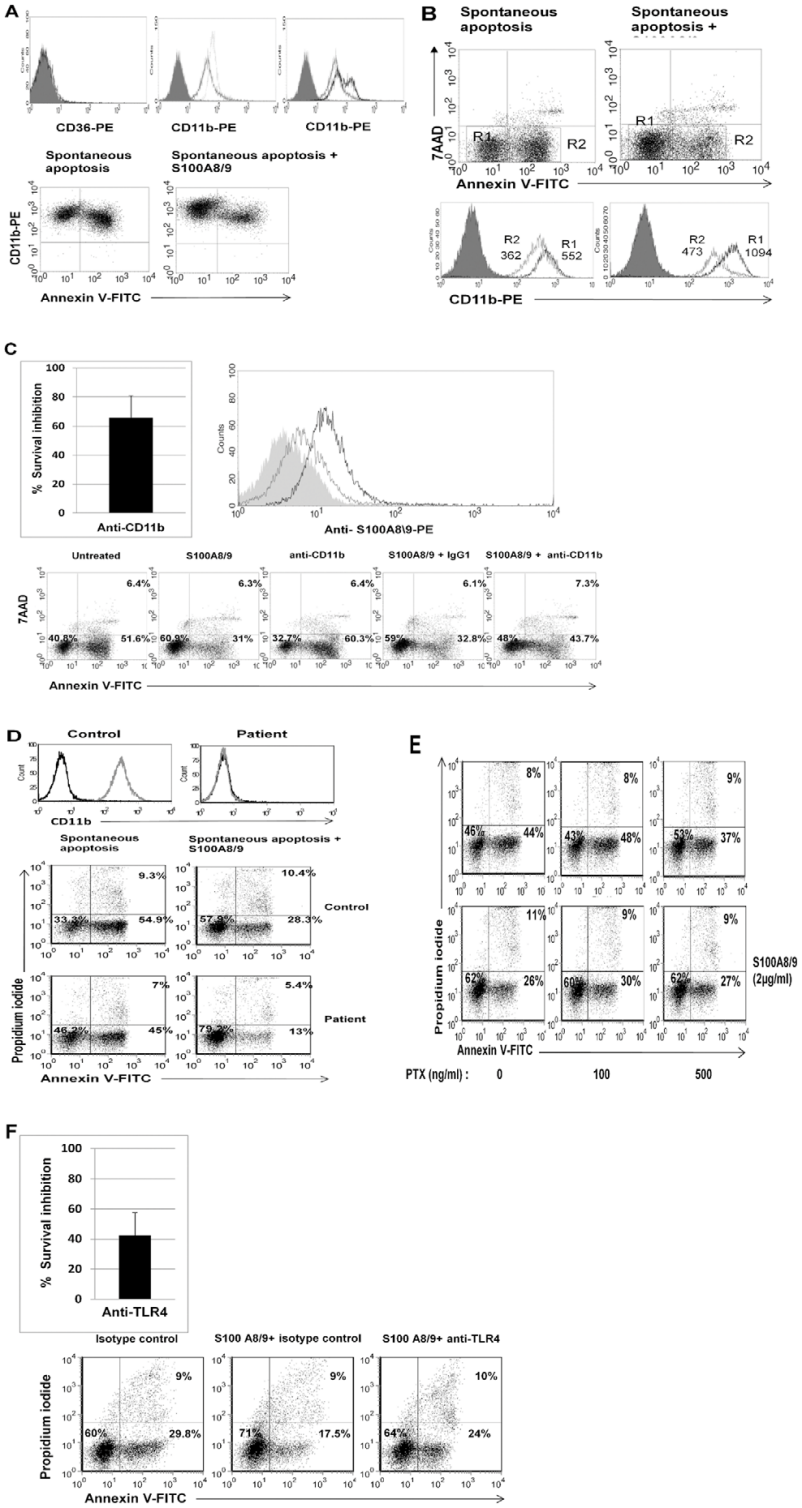
The survival effect of S100A8/9 is mediated through CD11b/CD18 and TLR4. **A.** Expression of CD11b on neutrophils. Upper panel. Freshly isolated neutrophils express CD11b/CD18 (isotype control is shown as filled histogram and anti- CD11b/CD18-PE in dotted line, middle) but not CD36 (left). Following 12 h of spontaneous PCD (gray line, middle) there was a marked decrease in the expression of CD11b/CD18 in comparison to freshly isolated neutrophils. Addition of S100A8/9 resulted in two different cell populations: high and low-CD11b/CD18 (black line, right). Lower panel. Left. Correlation between CD11b and phosphatidylserine expression demonstrates that when neutrophils become apoptotic they downregulate CD11b expression. Right. S100A8/9 upregulates CD11b expression. **B.** S100A8/9 dramatically increases CD11b on viable cells. Viable cells (R1, black line) increase CD11b expression from a mean fluorescence of 552 to 1094 (*p*<0.0001). Apoptotic cells (R2, gray line) still maintain some of this effect and show a mean fluorescence of 473, compared to 362 in the absence of S100A8/9. Filled histograms represent isotype control. Neutrophils were harvested after 12 h spontaneous constitutive PCD, with or without treatment with 2 µg/ml S100A8/9. Histograms are representative of at least 3 different experiments. **C.** The survival effect of S100A8/9 in the presence of anti-CD11b/CD18. Upper panel. Right. Neutrophils were incubated for 12 h and treated with either anti-CD11b or the isotype control IgG1 before addition of S100A8/9 complex. Integrin inhibition reduced the effect of S100A8/9 by 40−100% in comparison with the isotype control (*p*<0.001). The average of 6 experiments is presented. Lower panel. A representative dot plot is shown. Upper panel. Left. S100A8/9 binds to CD11b/CD18. CHO cells transfected with CR3 (black) or vector (gray) were evaluated for S100A8/9 binding. CR3-ransfected cells bound at rates almost twofold higher than CHO control cells (median fluorescence 13.3 vs. 6.6, *p*<0.001). The filled curve represents isotype control; the histogram is representative of 3 experiments. **D.** S100A8/9 effect on neutrophils from a CD11b/CD18-deficient patient. Neutrophil expression of CD11b from a healthy control and a patient with leukocyte adhesion deficiency is shown in the upper panel. Spontaneous constitutive apoptosis is shown in the lower panel, together with the S100A8/9 effect. Neutrophils were isolated from a CD11b-deficient patient and a healthy control, as described in Materials and methods, incubated at a concentration of 1×10^6^/ml and allowed to undergo spontaneous constitutive PCD for 10 h, with or without addition of S100A8/9. Apoptosis was assessed by Annexin V-PI staining. **E.** The survival effect induced by the complex S100A8/9 in the presence of pertussis toxin. *Bordetella pertussis* toxin at 100 and 500 ng/ml were added to neutrophils 45 min before adding S100A8/9. Neutrophils then were allowed to undergo constitutive spontaneous PCD that was assessed using AnnexinV-FITC and PI staining after 12 h. The dot plots are representative of 3 experiments. **F.** The survival effect of S100A8/9 in the presence of anti-TLR4. Neutrophils were incubated for 10 h and treated with either anti-TLR4 or the isotype control IgG2a, as described in Materials and methods. Inhibition of TLR4 abrogated the effect of S100A8/9 by 30−60% (*p*<0.001) in comparison with the isotype control, as seen in upper panel. The upper panel is presented as percentage of control. The experiment is a summary of four experiments (upper panel) with representative Plots in the lower panel.

S100A8 and A9 specific receptors have not yet been identified. However, Vogl et al demonstrated that S100A8 specifically interacts with the TLR4-MD2 complex [29], and Newton RA et al suggested that a pertusis toxin-sensitive G-coupled protein receptor could be the S100A9 receptor [30]. S100A8 and A9 have been shown to exert their effects via CD36 and CD11b/CD18 (Mac-1) [Bibr pone.0029333-Newton1] [Bibr pone.0029333-Kerkhoff1] [30−32].CD11b/CD18 is well expressed in freshly isolated neutrophils, but CD36 is not (Fig. 5A, upper panel). Furthermore, CD11b/CD18 was suggested as a pro-apoptotic integrin [33], [34].

Following spontaneous constitutive PCD, we detected a homogeneous decrease in neutrophil expression of CD11b/CD18. Gating apoptotic (Annexin V-positive) versus nonapoptotic (Annexin V-negative) cells (Fig. 5B) revealed higher CD11b/CD18 expression on viable- versus apoptotic neutrophils (Fig. 5A−B). Furthermore, adding S100A8/9 dramatically upregulated CD11b/CD18, establishing a new subpopulation with very high expression on viable cells (Fig. 5A−B).

We then blocked CD11b/CD18 prior to addition of S100A8/9. CD11b inhibition during constitutive spontaneous neutrophil PCD decreased cell survival by 20%, and in the presence of S100A8/9, CD11b inhibition cut the survival effect of S100A8/9 by 50−75% (Fig. 5C), suggesting that S100A8/9 may exert its survival effect, at least in part, by either binding or expressing CD11b, a known receptor involved in neutrophil apoptosis.

For further assessments, we used a CHO cell line transfected with CD11b/CD18. Transfected CHO cells increased binding of the S100A8/9 complex by almost twofold in comparison with control CHO cells (Fig. 5C, upper right). However, when we examined the effect of recombinant S100A8/9 on CD11b/CD18-deficient neutrophils from a leukocyte adhesion deficiency (LAD) patient, results were less clear. As expected, CD11b-deficient neutrophils showed delayed PCD in comparison with neutrophils from healthy donors (Fig. 5D), however the S100A8/9 complex kept its protective anti-apoptotic effect on CD11b-deficient neutrophils, and survival was increased by 50−90%. This supports the hypothesis that these proteins exert their survival effect only partially by CD11b/CD18, and other receptors are involved. To evaluate the role of one alternative candidate, we examined a G-coupled protein receptor. Pertusis toxin, which abrogates G-coupled protein receptor function, did not influence the survival effect of S100A8/9 (Fig. 5E). An additional possible receptor is Toll-like receptor 4 [29]. Inhibiting TLR4 abrogated the survival effect of S100A8/9 by 25−60% (Fig. 5F), suggesting that both CD11b/CD18 and TLR4 may be involved in the pathway enabling these proteins to affect neutrophil PCD.

**Figure 6:**
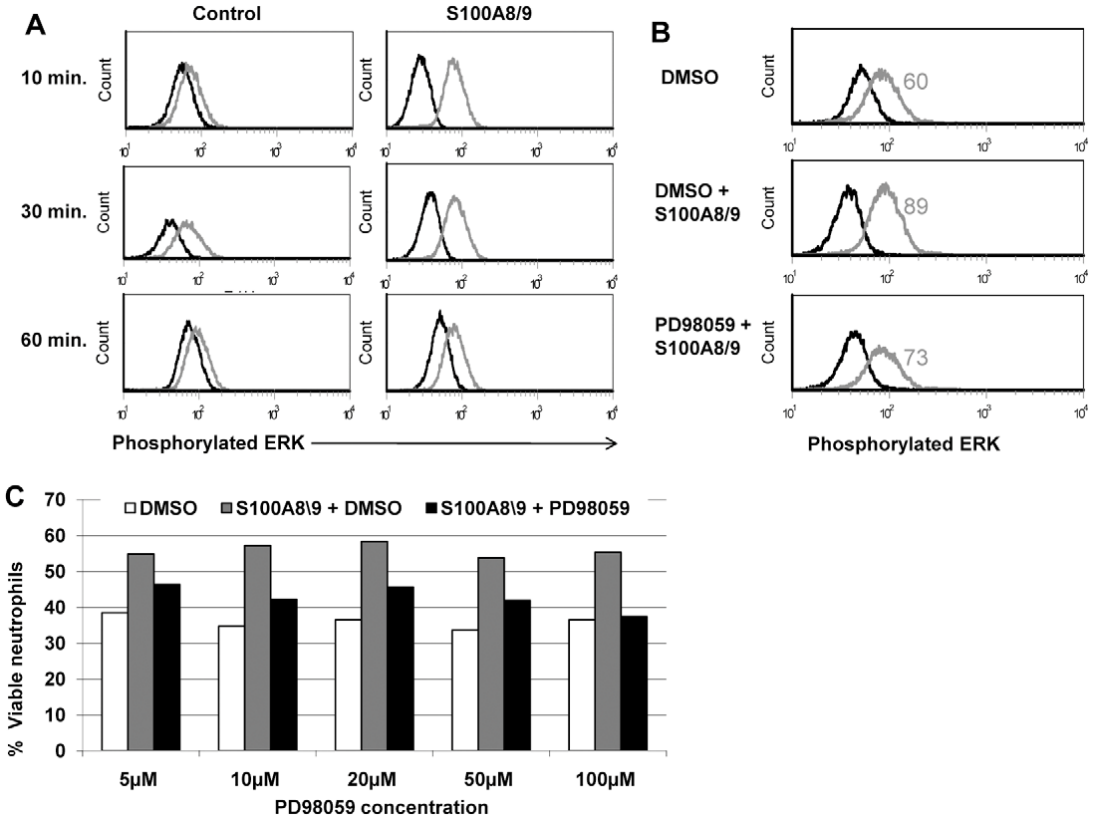
Effects of the S100A8/9 complex are mediated through the MAPK-ERK pathway. **A.** Phosphorylation of MAPK following exposure to S100A8/9. Freshly isolated neutrophils with or without treatment with 2 µg/ml S100A8/9 for various times as indicated, and then prepared for intracellular staining with mouse anti-human phospho-p44/42 MAPK alexa fluor 488 (gray) or isotype control (black), as described in Materials and methods. In the presence of S100A8/9, phosphorylation was increased by 3.22-fold after 10 min (*p*<0.001), 1.5-fold after 30 min (*p*<0.001), and decayed at 60 min (*p*<0.001). Histograms are representative of 3 independent experiments. **B.** Reduced phosphorylation of MAPK following exposure to S100A8/9 in the presence of PD98059, a MAPK phosphorylation inhibitor. Freshly isolated neutrophils were treated with the inhibitor PD98059 or with DMSO as a control for 30 min, and then treated with 2 µg/ml S100A8/9. Cells were then harvested and prepared for intracellular staining of phospho-p44/42 MAPK (gray) or isotype control (black), as described in Materials and methods. Treatment with PD98059 inhibitor reduced phosphorylation caused by the S100A8/9 complex (*p*<0.001). Histograms are representative of 3 experiments. **C.** Effect of the S100A8/9 complex in the presence of MAPK-ERK inhibitor. The effect of S100A8/9 was assessed in the presence of PD98059. Freshly isolated neutrophils were treated with varying combinations and concentrations of DMSO (white bars), S100A8/9 complex (gray bars), or S100A8/9 complex with PD98059 (black bars), as indicated, for 30 min before adding S100A8/9. Neutrophils were allowed to undergo constitutive spontaneous PCD. Results represent the percentage of viable cells according to Annexin V-FITC and PI staining. Data is representative of 3 experiments (*p*<0.001).

Extracellular signal-regulated kinase (ERK), one of the mitogen-activated protein kinases (MAPK), is involved in integrin signaling and Toll-like receptors in general. It appears to mediate signals promoting cell proliferation, differentiation, and survival, as reviewed by Roux, et al [35]. We assessed the role of ERK in the neutrophil S100A8/9 survival effect using intracellular staining of phosphorylated ERK. Neutrophils exposed to S100A8/9 for 10 min showed more than a threefold increase in phosphorylation of the ERK kinase (Fig. 6A). This activation continues to a lesser extent following 30 and 60 min exposure. Furthermore, the inhibitor PD98059 (MEK inhibitor) reduced ERK phosphorylation by more than 50% (Fig. 6B) and abolished the S100A8/9 survival effect by 50−100% at various concentrations (Fig. 6C), suggesting that the S100A8/9 complex survival effect involves the MEK-ERK signaling pathway.

### Apoptotic neutrophils are not activated neutrophils

As S100A8/9 was mainly reported thus far to be released during inflammation (see discussion), it may be attributed to release due to neutrophil activation. We wanted to further verify that apoptotic neutrophils are not activated due to extraction method or in vitro effect. Therefore we analyzed three surface molecules that are upregulated upon neutrophil activation and downregulated upon neutrophil PCD. In figures S1 and S2, CD16 and CD62L, both markers of neutrophil activation showed clear down regulation in the examined neutrophils, in correlation to apoptotic state [36]. In addition CD11b, is a third molecule, that is upregulated upon activation was downregulated in apoptotic neutrophils releasing S100A8 and S100A9 (see Figure 5). Taken together, S100 A8 and S100A9 were released in the context of apoptotic neutrophil death in non activated neutrophils.

## Discussion

Neutrophils are responsible for the body's rapid and effective response to infection or injury [37]. Efficient function necessitates an optimal balance between a sufficient number of blood neutrophils, response to stimuli, and transmigration properties, on one hand, against regulation of the termination of a neutrophil-mediated inflammatory response on the other. Thus, control of neutrophil turnover in the bone marrow and circulation, and at inflammatory sites, is key to both homeostasis and inflammation.

PCD plays a critical role in neutrophil homeostasis for an optimal functioning in the human body. Polymorphonuclears comprise approximately 75% of all nucleated cells in the hematopoietic compartment of the newborn marrow, with the majority being neutrophils. Already in the bone marrow, large-scale neutrophil death and removal of neutrophils by phagocytes are seen [38].Yet, the major regulator of blood neutrophil counts, as well as neutrophil counts at inflammatory sites, is PCD, which allows only a short life span, with modulation according to needs such as inflammation.

Here we show that the S100 A8/9 complex regulates neutrophil number and PCD in response to neutrophil concentration, by an anti-apoptotic effect. S100A9 (MRP14) and S100A8 (MRP8) constitute roughly 40% of the cytosolic protein in neutrophils; their pure abundance implies an important role in neutrophil functions. However, our understanding of the mechanisms implicating both molecules in neutrophil/monocyte physiology remains incomplete, as reviewed by Nacken, et al [39].

S100A9 and S100A8 readily form hetero- and homodimeric, trimeric, and tetrameric complexes, but apparently also exert specific functions as monomers. It has been suggested that S100A9 plays a prominent role in leukocyte trafficking and arachidonic acid metabolism, and elevated S100A9 and S100A8 levels are found in body fluids of inflamed tissues. When neutrophils undergo PCD, they release S100A8/9, which partially protects surrounding neutrophils from PCD, a mechanism that may be of crucial importance in fine-tuning neutrophil concentrations in the bone marrow and blood, and in inflammatory sites. This finding is supported by the decrease in bone marrow neutrophils in S100A9-deficient mice [40], and the accumulation of S100A8/9 at inflammatory sites, where prolonged survival of neutrophils is usually needed for chemotaxis and neutrophil adhesion [32].

As shown in figure 5A and supplemental figures 1 and 2, apoptotic neutrophils were not activated and their S100 A8 and S100 A9 release was associated with PCD and not inflammation or activation.

S100A8 deficiency is lethal [41], thus further research to gain understanding of the role of S100A8/9 is partially limited to animal models. By prolonging neutrophil life, S100A8/9 accumulation could be also a contributing mechanism to autoimmune conditions and the development of chronic inflammation or acute sterile danger-related inflammation. In conditions such as gout, monosodium urate monohydrate crystals induce the release of S100A8/A9 from neutrophils [42] and allow inflammatory attack by prolonging neutrophil life. High S100A8/9 levels were found in chronically inflamed joints of patients with rheumatoid arthritis [43].

Our study also proposes an additional possible mechanism for neutropenia following chemotherapy, suggesting that low neutrophil concentration may be further aggravated due to accelerated PCD, induced at low neutrophil concentrations. This hypothesis needs further investigation.

We were also able to gain a better understanding of the mechanism enabling S100A8/9 function. S100A8/9 clearly upregulates and binds to CD11b, previously shown to be a major regulator of neutrophil PCD [44], with possible anti- or pro-apoptotic effect. However, as shown here, this effect was retained in CD11b-deficient neutrophils, indicating additional mechanisms such as TLR4.

Indeed, blocking TLR4 partially abrogated the S100A8/9 survival effect, suggesting that S100A8/9 is an endogenous TLR4 ligand mediating survival. It has been shown that the TLR4 agonist LPS and others inhibit neutrophil apoptosis [12], [45]. Here we show for the first time an endogenous ligand that inhibits neutrophil apoptosis.

Our results also show a role for the MEK/ERK pathway following activation by S100A8/9. This pathway has been well documented to mediate signaling of both integrins and Toll-like receptors, supporting a role for CD11b, TLR4, and possibly additional receptors. This mechanism has been shown in the past to be anti-apoptotic in neutrophils via both intrinsic [46] and extrinsic [47] PCD pathways.

The correlation between beta2-integrin and TLR4 has been suggested. These two receptors may cooperate to produce signals that are transmitted into the cell. Also, TLR4 has been suggested to be an “inside-out” regulator of the beta2-integrins [48], and even a direct TLR4-beta2 integrin interaction has been suggested [49].

Other possible functional proteins that could have a “community effect” may be found in the secreted proteome of apoptotic cells. An experimental approach using 2D gel may discover additional candidate proteins and functional studies may establish their potential role in PCD.

In summary, the physiological role of S100A8/9, the most abundant cytosol protein in neutrophils, was not previously understood. We propose that at physiological protein [50] and cell concentrations, its main function is in modulating neutrophil PCD, and that cell concentration is regulated by an autocrine and paracrine mechanism via S100A8/9.

Finally, a biological “proof” is presented here, suggesting that nature encourages “community life” at the level of neutrophil survival, which could be seen as a cellular illustration for human life.

## Supporting Information

Figure S1 **Apoptotic neutrophils downregulate CD16.** The expression of CD16 (Fc gamma RIII) on neutrophils. Freshly isolated neutrophils CD16 expession at time 0 (black line, median fluorescence of 382) and following 12 h of spontaneous PCD (gray line, median fluorescence of 35). Isotype control is shown as filled histogram.
(TIF)

Figure S2 **Apoptotic neutrophils downregulate CD62L.** The expression of CD62L (L-selectin) on neutrophils. Freshly isolated neutrophils CD62L expession at time 0 (black line, median fluorescence of 791) and following 12 h of spontaneous PCD (gray line, median fluorescence of 27). Isotype control is shown as filled histogram.
(TIF)

Figure S3 **Apoptosis-related proteins during spontaneous constitutive PCD in the presence of S100A8/9.** SDS-PAGE of McL1, Bcl-xl, A1, Bcl2, and survivin in spontaneous constitutive apoptosis, with and without addition of S100A8/9. McL1 (39 KD) and Bcl-xl (26 KD) were detected in all conditions. A1 (20KD) was detected from freshly isolated neutrophils but expression was downregulated significantly by spontaneous constitutive apoptosis. Addition of S100A8/9 did not rescue protein expression. Bcl2 (25KD) and survivin (19KD) were not detected under any experimental conditions. The lysates of 40×10^6^ neutrophils under different conditions, including freshly isolated, after 8–10 h of spontaneous constitutive PCD, or after 8–10 h of spontaneous constitutive PCD with addition of S100A8/9, were loaded and separated by SDS-PAGE, as described in Experimental Procedures. Proteins were transferred to the PVDF membrane and exposed to the appropriate primary antibody according to manufacturers' instructions, and then to secondary antibody conjugated with HRP.
(TIF)

Methods S1 Additional details of methods used in proteomics and programmed cell death evaluation.
(DOC)
