# Supplementary figures and images for: Constitutive Neutrophil Apoptosis: Regulation by Cell Concentration via S100 A8/9 and the MEK – ERK Pathway
Source: PLoS One. 2012 Feb 17;7(2):e29333. doi: 10.1371/journal.pone.0029333 (PMC3281816; doi:10.1371/journal.pone.0029333)

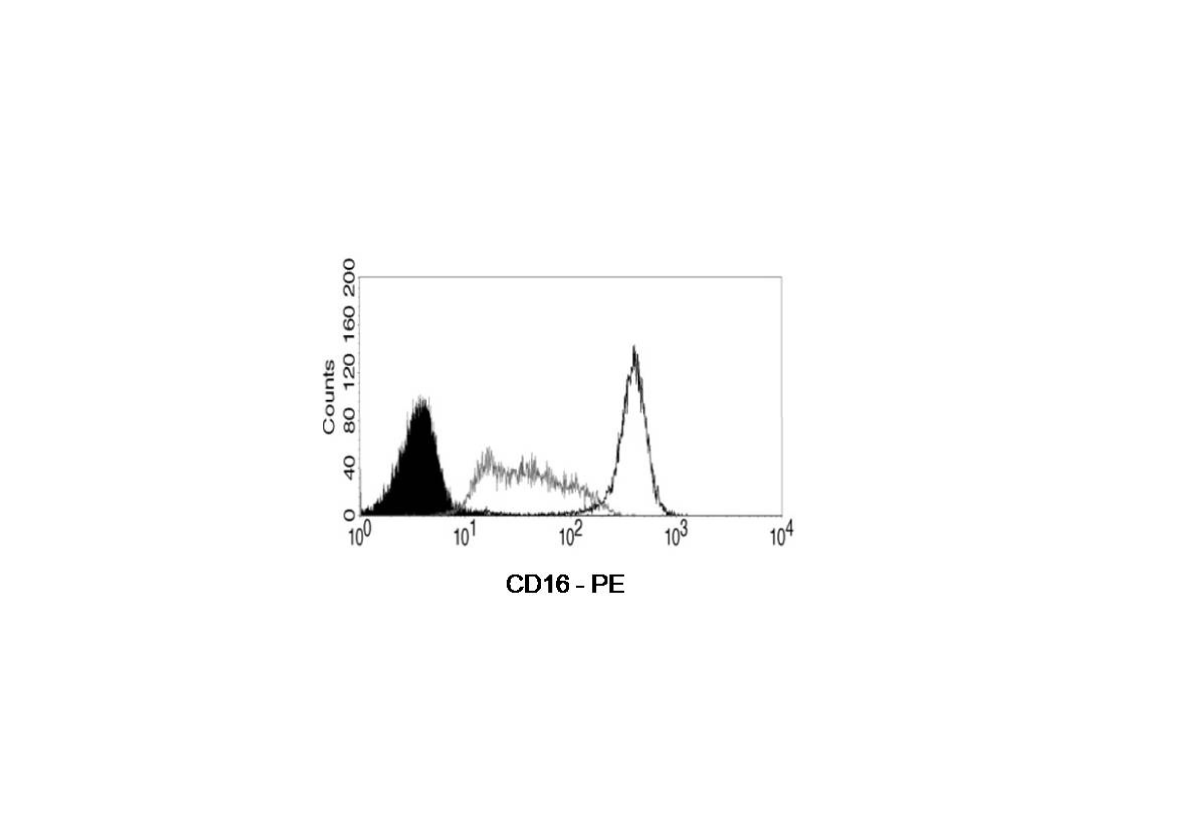

Supplement: Figure S1 — Apoptotic neutrophils downregulate CD16. The expression of CD16 (Fc gamma RIII) on neutrophils. Freshly isolated neutrophils CD16 expession at time 0 (black line, median fluorescence of 382) and following 12 h of spontaneous PCD (gray line, median fluorescence of 35). Isotype control is shown as filled histogram. (TIF) [file pone.0029333.s001.tif]

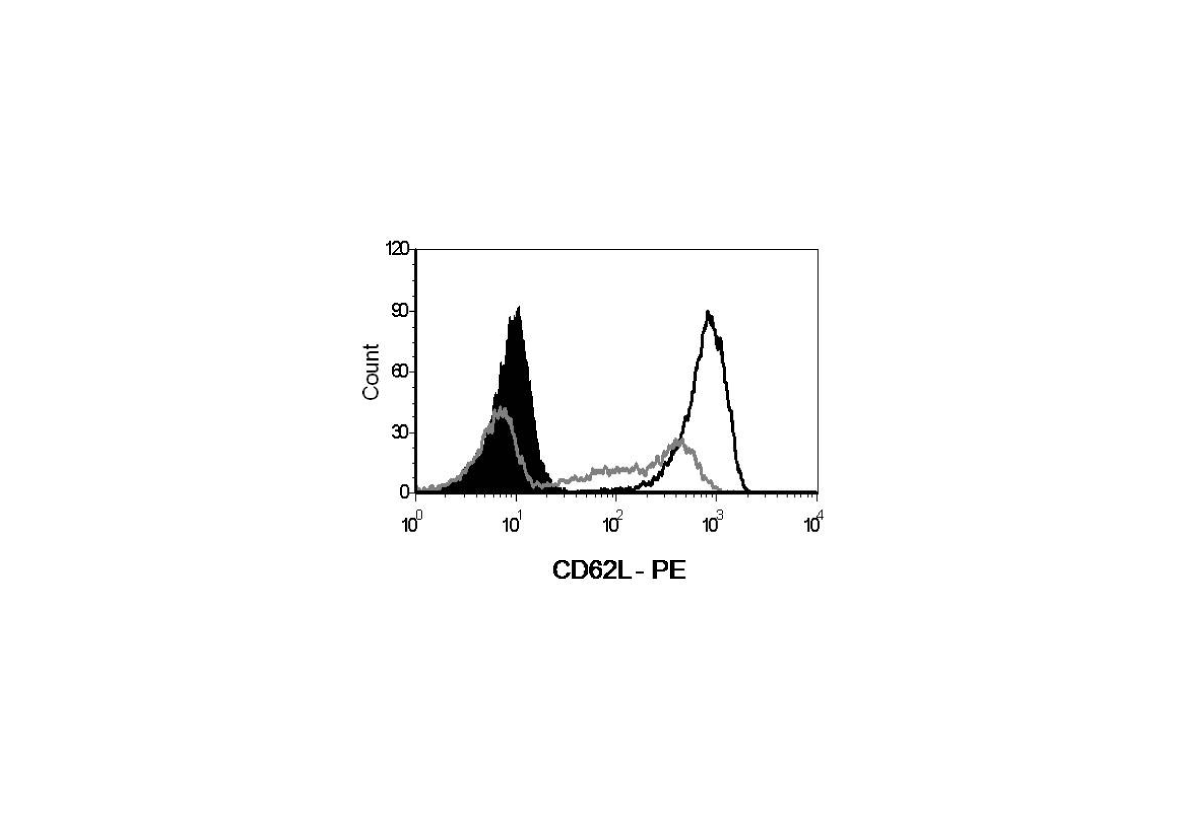

Supplement: Figure S2 — Apoptotic neutrophils downregulate CD62L. The expression of CD62L (L-selectin) on neutrophils. Freshly isolated neutrophils CD62L expession at time 0 (black line, median fluorescence of 791) and following 12 h of spontaneous PCD (gray line, median fluorescence of 27). Isotype control is shown as filled histogram. (TIF) [file pone.0029333.s002.tif]

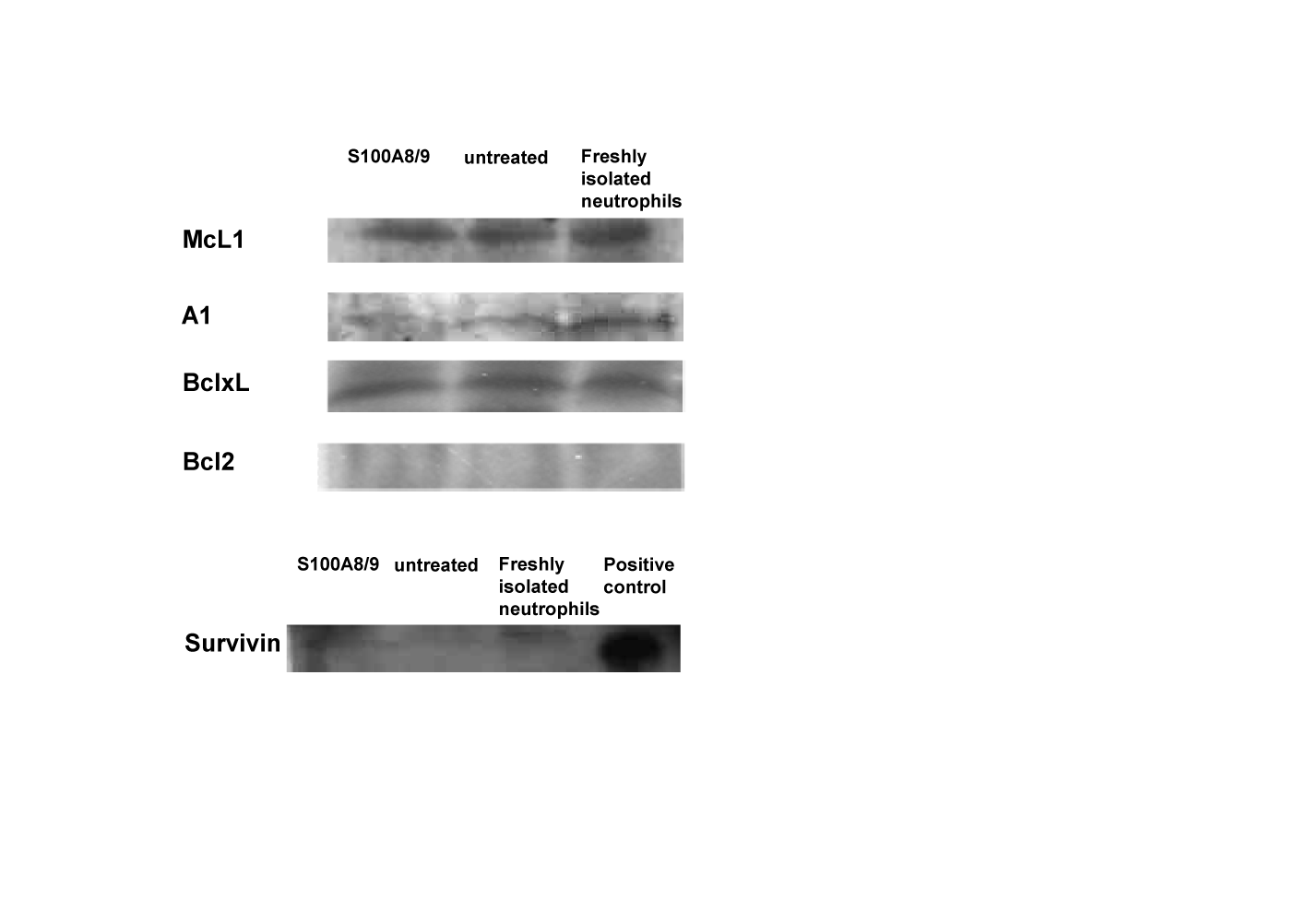

Supplement: Figure S3 — Apoptosis-related proteins during spontaneous constitutive PCD in the presence of S100A8/9. SDS-PAGE of McL1, Bcl-xl, A1, Bcl2, and survivin in spontaneous constitutive apoptosis, with and without addition of S100A8/9. McL1 (39 KD) and Bcl-xl (26 KD) were detected in all conditions. A1 (20KD) was detected from freshly isolated neutrophils but expression was downregulated significantly by spontaneous constitutive apoptosis. Addition of S100A8/9 did not rescue protein expression. Bcl2 (25KD) and survivin (19KD) were not detected under any experimental conditions. The lysates of 40×106 neutrophils under different conditions, including freshly isolated, after 8–10 h of spontaneous constitutive PCD, or after 8–10 h of spontaneous constitutive PCD with addition of S100A8/9, were loaded and separated by SDS-PAGE, as described in Experimental Procedures. Proteins were transferred to the PVDF membrane and exposed to the appropriate primary antibody according to manufacturers' instructions, and then to secondary antibody conjugated with HRP. (TIF) [file pone.0029333.s003.tif]
