# Supplementary material for: Constitutive Neutrophil Apoptosis: Regulation by Cell Concentration via S100 A8/9 and the MEK – ERK Pathway
Source: PLoS One. 2012 Feb 17;7(2):e29333. doi: 10.1371/journal.pone.0029333 (PMC3281816; doi:10.1371/journal.pone.0029333)
Supplement: Methods S1 — Additional details of methods used in proteomics and programmed cell death evaluation. (DOC) [file pone.0029333.s004.doc]

**Supporting information**

**Methods S2**

**Apoptosis assessment**

Cell viability was determined by Trypan blue exclusion using a light microscope, at varying times after incubation. PCD of neutrophils was determined in several ways. Phosphatidylserine exposure was determined by measurement of Annexin V- FITC binding, and cells were also stained with PI for detection of necrotic cells. The stain was made as described previously [27]. When indicated, nucleic acid dye 7-amino-actinomycin D (7AAD, BD Biosciences Pharmingen, San Diego, CA) replaced PI, since 7AAD used in conjunction with phycoerythrin (PE)- provides monoclonal antibody and fluorescein isothiocyanate (FITC) labeling in three-color analysis, with minimal spectral overlap between 7-AAD, PE, and FITC fluorescence emissions. Cells (1x10^4^) were then analyzed using CellQuest software (Becton Dickinson, Mountain View, CA). Mitochondrial permeability transition was detected and used as another method for assessment of apoptosis, based on the ability of intact mitochondria to take up and retain cationic lipophilic fluorescent dyes. Neutrophils were loaded with 5-15 nM DiOC6 for 30' at 37°C. Cells were then stained with PI for 10 min, resuspended in ice-cold PBS, and analyzed by flow cytometry.

**Proteomic (SDS-PAGE and MS)**

***I.* *Sample preparation***

Neutrophil culture media, cultivated at low- (0.5x10^6^/ml) or high (16x10^6^/ml) concentrations, was collected and compared using SDS-PAGE. Culture media was cleared of cells and undesired cell debris by sequential centrifugations – first at 1,200 RPM for 10 min, then 14,000 xg for 15 min, and finally 55,000 xg for 1 h – using a Beckman Ti100 centrifuge with a TLS55 rotor (Beckman Coulter, Krefeld, Germany). The resulting supernatant was collected and analyzed. Prior to electrophoresis, proteins were concentrated and desalted using Sep-Pak C-18 cartridges (Waters Corporation, Milford MA). Protein concentration was determined using the Bradford assay (BioRad, Hercules CA).

***II. SDS-PAGE***

Gradient 4-20% polyacrylamide-SDS gels and SDS buffer were prepared. The molecular mass of the protein bands was determined with a Precision Plus Protein Standards Kit (Bio-Rad). Proteins were visualized using the Bio-Safe Coomassie (Bio-Rad) according to the manufacturer's instructions. Gel images were acquired using a Power Look III Scanner (Umax, Dallas, TX).

***III. ESI-MS/MS***

Nano-Electrospray ionization tandem mass spectrometry was carried out at the mass spectrometry facility in the Interdepartmental Unit of the Hebrew University-Hadassah School of Medicine. For Trypsin digestion, proteins from concentrated medium fractions were separated by SDS-PAGE. The region corresponding to the differential protein was excised and subjected to an in-gel digestion procedure. Briefly, the procedure includes washing and drying of gels, reduction and alkylation, rehydration with 10ng/ml Trypsin in 25mM ammonium bicarbonate buffer solution, incubation for 12-16 h at 37°C, and peptide extraction. In-gel tryptic digests were further desalted using C18 ZipTips (Millipore, Billerica, MA), and eluted in 5 µl of an elution buffer containing 60% (v/v) acetonitrile in 0.1% (v/v) formic acid (Mallinckrodt Baker, Phillipsburg, NJ).

Mass spectrometry was performed using a Micromass Q-Tof system, equipped with a NanoFlow Probe Tip Type F (Micromass UK Ltd., Manchester, UK). The extracted peptide solution was collected in a borosilicate capillary tip (Protana, Odense, Denmark) and subjected to electrospray ionization (ESI) at a flow rate of 10 nl/min. MS spectra were analyzed with MicroMass Protein Lynx software. Protein identification was conducted using the MS-FIT proteomic tool from the Matrix-Science web server.

**Western blotting**

Rabbit polyclonal antibody against human Mcl-1 (Becton Dickinson, Franklin Lakes, NJ), rabbit polyclonal antibody against human survivin (R&D Systems, Minneapolis, MN), mouse monoclonal anti-human Bcl2, mouse monoclonal anti-human Bcl-xl (Calbiochem, San Diego, CA), and rabbit polyclonal anti human A1 (Santa Cruz Biotechnology, Santa Cruz, CA) were used to detect the proteins by immunoblotting. Freshly isolated neutrophils (40 x 10^6^) and neutrophils undergoing constitutive spontaneous PCD were lysed and loaded on 14% SDS-PAGE gel, transferred to a PVDF membrane (Millipore), and blocked with 20% skimmed milk in PBST (PBS*1, 0.05-0.1% Tween 20). The membrane was incubated with primary antibody overnight at 4°C, then washed with PBST, and incubated for 30 minutes with 1:10,000 HRP-conjugated goat anti-mouse or goat anti-rabbit secondary antibody. Proteins were visualized with the EZ-ECL detection kit (Biological Industries, Beit Haemek, Israel)
